# Viral Etiology of Respiratory Tract Infections in Children at the Pediatric Hospital in Ouagadougou (Burkina Faso)

**DOI:** 10.1371/journal.pone.0110435

**Published:** 2014-10-31

**Authors:** Solange Ouédraogo, Blaise Traoré, Zah Ange Brice Nene Bi, Firmin Tiandama Yonli, Donatien Kima, Pierre Bonané, Lassané Congo, Rasmata Ouédraogo Traoré, Diarra Yé, Christophe Marguet, Jean-Christophe Plantier, Astrid Vabret, Marie Gueudin

**Affiliations:** 1 Charles de Gaulle Pediatric University Hospital, Ouagadougou, Burkina Faso; 2 Respiratory Diseases, Allergy and CF Unit, Paediatric Department, Rouen University Hospital Charles Nicolle, EA3830, Inserm CIC204, Rouen, France; 3 Laboratory of Virology, GRAM EA 2656 Rouen University Hospital Charles Nicolle, Rouen, France; 4 Laboratory of Human and Molecular Virology, Caen University Hospital Clemenceau, Caen, France; CEA, France

## Abstract

**Background:**

Acute respiratory infections (ARIs) are a major cause of morbidity and mortality in children in Africa. The circulation of viruses classically implicated in ARIs is poorly known in Burkina Faso. The aim of this study was to identify the respiratory viruses present in children admitted to or consulting at the pediatric hospital in Ouagadougou.

**Methods:**

From July 2010 to July 2011, we tested nasal aspirates of 209 children with upper or lower respiratory infection for main respiratory viruses (respiratory syncytial virus (RSV), metapneumovirus, adenovirus, parainfluenza viruses 1, 2 and 3, influenza A, B and C, rhinovirus/enterovirus), by immunofluorescence locally in Ouagadougou, and by PCR in France. Bacteria have also been investigated in 97 samples.

**Results:**

153 children (73.2%) carried at least one virus and 175 viruses were detected. Rhinoviruses/enteroviruses were most frequently detected (rhinovirus n = 88; enterovirus n = 38) and were found to circulate throughout the year. An epidemic of RSV infections (n = 25) was identified in September/October, followed by an epidemic of influenza virus (n = 13), mostly H1N1pdm09. This epidemic occurred during the period of the year in which nighttime temperatures and humidity were at their lowest. Other viruses tested were detected only sporadically. Twenty-two viral co-infections were observed. Bacteria were detected in 29/97 samples with 22 viral/bacterial co-infections.

**Conclusions:**

This study, the first of its type in Burkina Faso, warrants further investigation to confirm the seasonality of RSV infection and to improve local diagnosis of influenza. The long-term objective is to optimize therapeutic management of infected children.

## Introduction

Respiratory viruses are ubiquitous, but most epidemiological knowledge relates to developed countries. In contrast, the burden of acute respiratory infections (ARIs) is particularly heavy among children in developing countries, with high mortality and hospital admission rates. The number of deaths related to ARIs has been estimated at 1.9 million children aged less than 5 years, 70% of whom live in Africa or South-East Asia [1]. In Burkina Faso (West Africa), ARIs are also a major cause of child admissions to hospital [2] with a 17.6% mortality rate in children aged under 5 years [3]. For a long time now, *Streptococcus pneumonia*, *Haemophilus influenza* and *Staphylococcus aureus* have been considered as the sole causal agents of severe ARIs in developing countries, and guidelines recommend prescribing antibiotics. Conversely, detection of viruses by molecular methods has provided evidence that a growing number of respiratory viruses are potent pathogenic agents for the respiratory tract. Thus, respiratory syncytial virus (RSV), human metapneumovirus (hMPV), rhinoviruses, parainfluenza (PIVs) and influenza viruses are currently recognized as common ARI etiologies in young children in developed countries [4], [5]. The etiology of ARI is complex and emphasized by the demonstration of viral, bacterial or mixed co-infections in the respiratory tracts [5], [6], [7].

However, in developing countries and especially in Africa, studies on virus-related ARIs are limited to very few countries. Nevertheless, the results of these studies confirm epidemiological data reported in developed countries and underline the fact that viruses also cause frequent upper or lower airway infections. Early diagnosis facilitates early management and is recognized as one way to combat ARIs [8]. In fact, lack of sensitivity and specificity of symptoms prevents differentiation between influenza or any other viral infection and malaria [9], [10]. The recommended early and easy use of antibiotics is not effective in viral ARIs, and can only prevent occurrence of bacterial super infection. In this context, viral diagnosis can prevent use of unnecessary costly antibiotics or antimalarial treatments.

To our knowledge, no specific data concerning the circulation of these viruses is currently available for Burkina Faso, even for influenza virus, which is the most documented virus internationally [11].

We carried out a prospective study, over a period of one year at Charles de Gaulle pediatric University Hospital in Ouagadougou (Burkina Faso). The aim was to determine the microbiological agents of these respiratory diseases using rapid detection of antigens by immunofluorescence, multiplex molecular tests, and bacterial cultures on nasopharyngeal samples. We aimed to improve our knowledge on the circulation of viruses and the type of ARIs that they cause.

## Materials and Methods

### Patients

This prospective study was conducted at Charles de Gaulle pediatric hospital in Ouagadougou, between July 1^st^ 2010 and June 30^th^ 2011. Inclusion criteria were as follows: Children aged under three years attending or hospitalized with an upper or lower airway infection. Upper respiratory infections were defined as congestive otitis, rhinitis associated with fever, and a hoarse cough suggesting tracheitis or laryngitis. Lower respiratory infection were defined as acute febrile respiratory distress, acute bronchiolitis, acute coughing or wheezing, febrile chest sounds suggesting pneumonia, bronchiolitis, asthma exacerbation. Oral parental consent was obtained to use a part of the nasopharyngeal aspiration for PCRs and clinical data were collected. In French law, the right to use the end of the samples is written in the code of public health: Code de la santé publique - Article L1211-2. The ethics committee in Burkina Faso was not consulted as it was recent when the study started.

### Detection of viruses

At the hospital laboratory in Ouagadougou, antigens for RSV, hMPV, influenza virus A and B, parainfluenza type 1, 2, and 3, and adenovirus were detected by direct immunofluorescence assay (DFA) from nasopharyngeal aspiration (NPA) samples, employing commercial monoclonal antibodies conjugated with fluorescein isothiocyanate (Imagen, Oxoid, UK). A positive result was indicated by DFA, if a technician noted presence of at least one cell showing a typical fluorescence pattern, provided that at least 20 respiratory cells were available in the sample. All the samples were frozen at −80°C and further analyzed by molecular methods at the virology laboratory of Caen University Hospital. Nucleic acids were extracted with a Qiasymphony kit (Qiasymphony Virus/Bacteria Minikit, Qiagen, Courtaboeuf, France), and RT-PCR was carried out for detection of RSV, hMPV, influenza A and B viruses (RSV/hMPV r-gene, Influenza A/B r-gene, Argène Biomérieux) and rhinovirus/enterovirus and influenza C virus (in-house multiplex RT-PCR) [12]. Viral subtyping was carried out according to the National Reference Center for Influenza techniques (Institut Pasteur, Paris, France).

### Bacterial growths

Bacteriological examinations were carried out only on 97 samples collected between the end of March and end of June 2011. NPA cultures were performed for growth of common and potentially pathogenic aerobic bacteria: *Streptococcus pneumoniae, Haemophilus influenzae, Moraxella catharralis, Staphylococcus aureus*, and *Klebsiella pneumonia*.

### Climatic data

Burkina Faso has a tropical Sudanian-Sahelian climate with two opposite seasons: a rainy season, with 300 to 1200 mm of precipitation, and a dry season characterized by the Harmattan, a hot dry wind loaded with dust and originating in the Sahara Desert. The data used were obtained from the meteorological archives of Ouagadougou Airport *(source* http://rp5.ru/archive.php?wmo_id=65503&lang=fr *)*. From daily data available, we calculated mean monthly values for temperature and humidity at 6 AM and 12 noon.

### Statistical analysis

MedCalc software was used for the comparison of rates realized in Table 1. A *P* value of 0.05 was considered statistically significant. For the climatic data, mean monthly values for humidity and temperature for the months with or without detection of RSV or influenza A virus were compared using t-test after verification of the equality of variances by a F-test.

**Table 1:** Respiratory viruses detected either by direct immunofluorescence or RT-PCR and the associated final diagnosis (in some cases, more than one), * significant difference (p = 0.0006) with the group where no virus was detected.

Virus and viral co-infection	N (%) results		Children under 1 year old	Children admitted to hospital	Pneumonia	Bronchiolitis	Bronchitis/Asthma	Laryngitis	Nasopharyngitis/Otitis
None	56	26.8%	37	38	8	6	26	3	28
Adenovirus only	2	1.0%	1	2		1	2		1
Adenovirus + Rhinovirus	1	0.5%	1	1					1
Influenza A only	8	3.8%	4	3	2		3	1	4
Influenza A + Enterovirus	1	0.5%	0	0					1
Influenza A + Rhinovirus	2	1.0%	2	2		2	1		1
Influenza A + Rhinovirus + Enterovirus	1	0.5%	0	0					1
Influenza C + Rhinovirus	1	0.5%	1	1		1			
Parainfluenza 1 + Rhinovirus	1	0.5%	0	0					1
Parainfluenza 1 + RSV	1	0.5%	1	0			1		1
Parainfluenza 2	1	0.5%	1	1			1		
Parainfluenza 3 + Enterovirus	4	1.9%	2	3	1	1	3		2
Parainfluenza 3 + Rhinovirus	1	0.5%	0	1		1			
RSV only	19	9.1%	14	14	6	10*	8		5
RSV + Rhinovirus	3	1.4%	2	3	1	2	1		
RSV + Enterovirus	2	1.0%	2	2		2	2		
Metapneumovirus	1	0.5%	1	0		1	1		
Enterovirus only	26	12.4%	12	18	3	2	11	1	18
Rhinovirus only	74	35.4%	43	44	8	11	35	3	37
Rhinovirus + Enterovirus	4	1.9%	3	3	1	2	1		3
**Total**	**209**	**100.0%**	**127**	**136**	**30**	**42**	**96**	**8**	**104**

## Results

Two hundred and nine children (boys: 58.4%) were included in this study. They were all aged less than three years, and 60.8% of them were less than 1 year old. Seventy-three children (34.9%) attended outpatient consultation, and 136 (65.1%) were admitted to hospital. Respiratory symptoms are described in Table 1.

One hundred and fifty-three children (73.2%) carried at least one virus (table 1). Children with positive results were mostly identified by RT-PCR (n = 149, 71.3%), and only 21 (10%) were detected by DFA. Positive results by DFA were as follows: adenovirus (*n* = 3), parainfluenza virus 1 (*n* = 2), parainfluenza virus 2 (*n* = 1), parainfluenza virus 3 (*n* = 5), and RSV (*n* = 10). RT-PCR detected: rhinovirus (n = 88; 59.1%), enterovirus (n = 38; 25.5%), RSV (n = 24; 16.1%), influenza (n = 13, including one case of influenza C; 8.7%), and one case of hMPV. Co-infections were detected in 14 samples (9.4%). Only one discordant result was observed for one sample, which was positive for RSV by DFA and negative by PCR. Among the viruses tested, only influenza B was never detected. Twenty-two (14.4%) viral co-infections were observed involving mainly rhinovirus or enteroviruses. Ninety eight (72.1%) of the inpatients carried at least one virus and 55 (75.3%) of the outpatients.

Bacterial cultures were carried out for 97 samples. Eighteen (18.6%) were negative for Bacteria and Viruses, 50 (51.5%) were positive for one or more viruses, 7 (7.2%) were positive for one or more bacteria and 22 (22.7%) were positive with a viral/bacterial co-infection. *Staphylococcus aureus, Klebsiella pneumonia*, and *Streptococcus pneumonia* were isolated in 14 cases (42.4%), 10 cases (30.3%), and 9 cases (27.7%) respectively.

Twenty-two viral/bacterial co-infections were diagnosed, 20 of which involved an enterovirus and/or a rhinovirus. The remaining two viral/bacterial co-infections associated *Staphylococcus aureus*/PIV-3 and *Staphylococcus aureus*/adenovirus. Four bacterial co-infections were detected: *Staphylococcus aureus*/*Klebsiella pneumoniae* (*n* = 2) and *Staphylococcus aureus/Streptococcus pneumoniae* (*n* = 2). Rhinovirus was also detected in three of these four cases. 76 of the 97 children (78.4%) were hospitalized and 26 (34.2%) of them were infected with a Bacteria.

**Figure 1:**
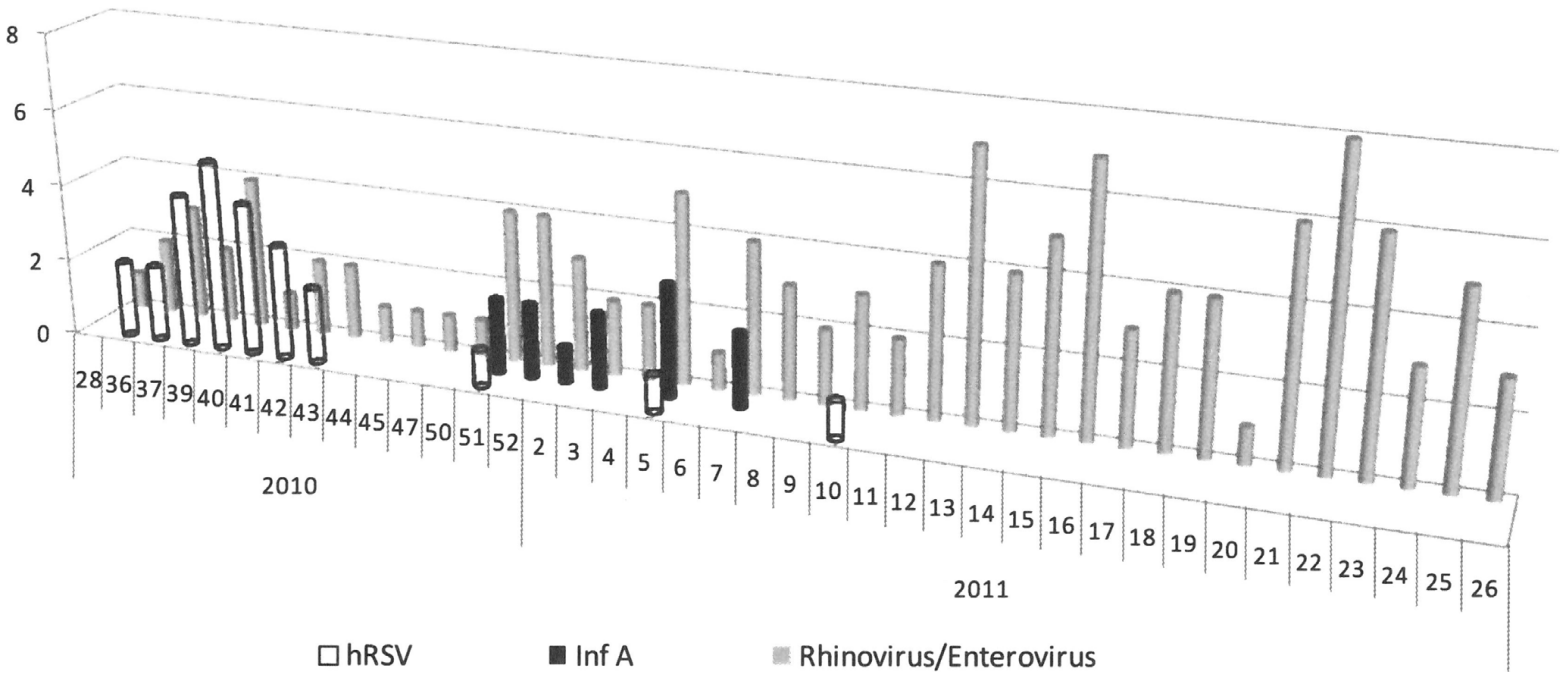
Weekly distribution of the three main detected viruses.

Monthly distribution and weekly detailed circulation of viruses (Figure 1 and Table 2), showed two successive winter epidemics. A RSV epidemic (24 RSV A, 1 RSV B) occurred between mid-September and end of October, with six co-infections including five rhinoviruses/enteroviruses. Nineteen of the 25 children infected with RSV were hospitalized which correspond to 14% of all the inpatients and 8% of the outpatients without significant difference (p = 0.2517). Children with RSV had significantly more bronchiolitis than the others (p = 0.0006) (table 1). RSV was found in the samples of 16 of the 70 children (22.9%) under the age of 6 months and in 9 of the 139 children above 6 months (6.5%) with significant difference (p = 0.0012).

**Table 2:** Monthly distribution of viruses.

Year/Month	AdV	Inf A	Inf C	hRSV	hMPV	PIV 1	PIV 2	PIV 3	RhV	EnV	Total	
2010/07									1		1	0.6%
2010/09				6					5		11	6.3%
2010/10				16	1				5	6	28	15.9%
2010/11									3		3	1.7%
2010/12		4		1		1			7	2	15	8.5%
2011/01		3							6	3	12	6.8%
2011/02	2	5		1					6	6	20	11.4%
2011/03				1		1		2	10	4	18	10.2%
2011/04	1							1	14	8	24	13.6%
2011/05								1	14	4	19	10.8%
2011/06			1				1	1	17	5	25	14.2%
	3	12	1	25	1	2	1	5	88	38	176	
	1.7%	6.8%	0.6%	14.2%	0.6%	1.1%	0.6%	2.8%	50.0%	21.6%		

This epidemic of RSV infection was followed by an epidemic of influenza A infection (H1N1pdm09, n = 8 and H3N2, n = 4) from mid-December to mid-February. These viruses were frequently associated with a rhinovirus or enterovirus (38.4%). Six children were hospitalized.

One hundred and nineteen children (56.9%) had a rhinovirus or an enterovirus which was detected during the year, with higher rates of rhinoviruses in April, May and June 2011. Rhinovirus and enterovirus were detected in 56.6% of the inpatients and 57.5% of the outpatients without significant difference (p = 0,8353). Of the 97 samples that underwent both bacteriological and virological investigation, 44 were positive for only enterovirus or human rhinovirus. A total of 56.5% of these children were under the age of 1 year and 39% were hospitalized.

Three adenovirus infections were detected at the end of February and five infections with parainfluenza virus 3 from March to June 2011. Only one of the three children infected with adenovirus was co-infected with a rhinovirus. The five children infected with parainfluenza virus 3 were co-infected with a rhinovirus or enterovirus and four of the five children were hospitalized.

**Figure 2:**
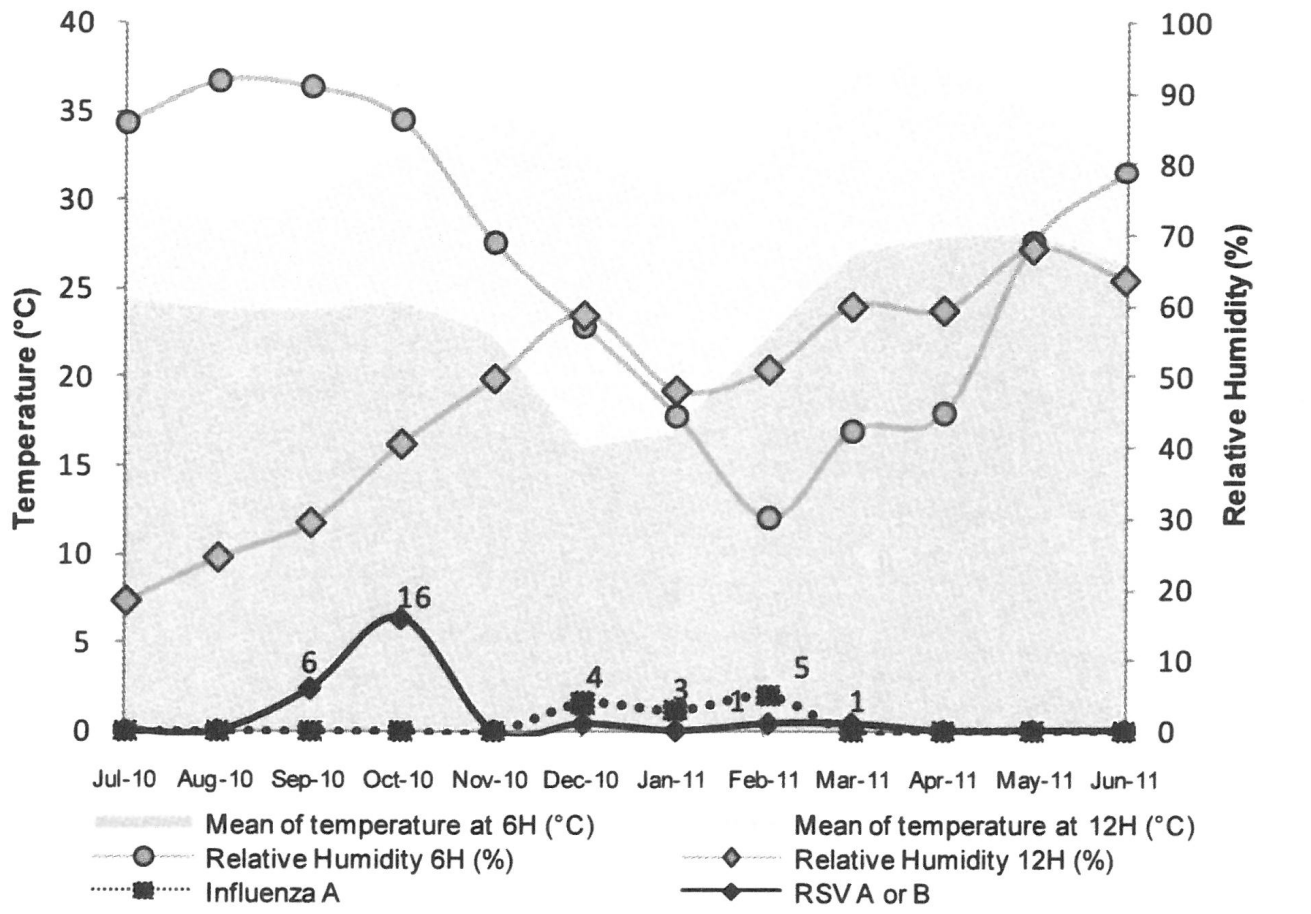
Progression of the epidemics of RSV and influenza virus infections related to temperature and relative humidity values recorded at 6 AM and 12 noon.

An effect of climate has often been put forward as an explanation for the circulation patterns of respiratory viruses. A comparison of our findings for influenza and RSV with the available climatic data (Figure 2) showed that the influenza epidemic coincided with the period in which nighttime temperature (p = 0.0007) and relative humidity (p = 0.0343) were lowest. No significant climatic data were related to RSV epidemic.

## Discussion

The epidemiology of respiratory virus infections is unknown in Burkina Faso, although suspected considering previous studies conducted in neighboring countries [13], [14]. Firstly, this study provides evidence of the role of viruses in upper and lower airway diseases in this country. Our findings highlight the fact that three quarters of infants or young children with any respiratory symptoms carry at least one virus. This rate of detection concords with previous studies in developed and African countries [15], [16], [17], [18]. Most of the viruses were identified by molecular diagnosis, and direct immunofluorescence yield was low but concordant with previous work [19], achieving only 10% of positive results. One discrepancy has been observed with a sample positive by DFA and negative by PCR without possible control. It has been verified that the exclusion of this sample would not have changed the statistical conclusions of the study. RSV must be considered as a main etiologic agent even if rhinoviruses or enteroviruses were most frequently detected. RSV is well known for being one of the main agents associated with upper or lower airway infections in infants, and it has been demonstrated that RSV causes more severe diseases than other respiratory viruses [6]. Although it is a universal virus, data on RSV circulation are still limited in Africa. In a previous review, *Stensballe et al.* suggested that RSV outbreaks begin on the southern coast and move northward from January to July [20]. In our study, the RSV outbreak in Burkina Faso was observed during the fall. This was consistent with data on RSV epidemics occurring in the fall or winter in neighboring countries like Senegal [13], Nigeria [21] and Cameroon [17]. In Ghana [14], another neighboring country to the south, RSV circulation was however observed throughout the year with a peak rate in summer. The influence of the climate remains difficult to demonstrate, and the various climates associated with RSV epidemics tend to prove the lack of impact of climate on their onset. In Burkina Faso, the peak for RSV infections coincided with the dry season, as reported in Nigeria and South Africa [21]. In contrast, RSV epidemics occur during the rainy season in Mozambique [21] and Ghana [14]. Lastly in Kenya the duration of RSV seasons was long, but there were no clear climate patterns that appeared to coincide with changes in RSV circulation [19], [22].

The second main finding of our study was the high rate of picornaviruses, and more than half of this population carried rhinoviruses or enteroviruses. The technique that we used for virus detection was not based on sequencing, and discrimination between the two species cannot be guaranteed. Nevertheless, it is clear that these viruses circulated throughout the year and were mainly isolated alone, although they are implicated in all but one viral co-infections. This high rate underlines the major involvement of these viruses in respiratory tract infections, as previously reported elsewhere in the world [23]. Picornaviruses are not the focus of the rare epidemiological studies conducted in Africa. They also appear to be the most frequent viruses identified in Cameroon, and Kenya [24], and a recent molecular study [25] detected all three (A, B and C) strains of rhinovirus in up to 35% of samples collected, as previously described in developing countries.

The bacteriological examinations were carried out during only 3 months, which is an important limitation for data analysis. During this period more than one quarter of this young population had a bacteria/virus co-infection. These co-infections associated an enterovirus or a rhinovirus with common potentially pathogenic bacteria encountered in childhood. When co-infections are identified, the respective contribution of each microbiological agent in the pathogenesis of respiratory tract infections remains unclear. Among the virus/virus co-infections, RSV was clearly identified as the most virulent agent, and its pathogenic effect predominated rhinovirus [6]. It is more difficult however to assess the respective pathogenic effect of bacteria or viruses detected simultaneously. Positive nasopharyngeal bacterial culture is a weak predictor of upper airway infections since healthy individuals often carry pathogenic bacteria. The presence of bacteria does not allow to conclude or to exclude to an isolated bacterial infection in upper airway infections. Conversely, Rhinovirus was detected as the sole causal agent in severe acute respiratory distress [24] and pneumonia [26] in Kenya and South Africa, respectively. This latter is in contrast with the better outcome attributed to Rhinovirus in developed countries [23], which is not necessarily associated with more severe disease.

Clinical diagnoses involving life-threatening infections such as malaria and meningitis were mentioned in the data collected. However, laboratory confirmation of these diagnoses was not part of our study and it was not possible to further analyze these cases.

Lastly, influenza A appeared as the fourth most prevalent virus in Ouagadougou, and was detected from mid December to the end of February. This result has not been compared with the Global Influenza Surveillance and Response System database, as no data on influenza epidemics was available from Burkina Faso for this season. This winter epidemic period of influenza A infection does not match that reported for neighboring countries. Nevertheless, this influenza epidemic occurred during the coldest period of the year, when relative humidity was low. This agreed with recent reports, showing that survival of the influenza virus in the external environment is related to low relative humidity and temperature [27], [28], [29]. No influenza B virus was identified in this study, contrasting with the high rates observed in neighboring countries over the same period. As our study mainly included very young children, for whom influenza virus was not reported as a major pathogenic agent [6], the likelihood of detecting either influenza A or B was reduced. Moreover, we are not able to rule out a concomitant circulation of influenza B in older populations.

Our study is the first of its type in Burkina Faso and warrants follow-up to confirm the seasonality of RSV infection and the period during which this virus circulates. The results presented here assess the involvement of the most frequent pathogenic viruses in upper and lower respiratory tract infections in young children from Burkina Faso. Our findings raise the question of sparing antibiotics by introducing routine detection of viruses. Such a strategy would be supported by the weak mortality attributed to RSV, which is the most aggressive viral agent in Africa [21]. However, the expensive cost of multiplex PCR testing prevents feasibility in routine practice. The less costly direct immunofluorescence assay has shown very low yield in this study, and consequently can not be recommended as an alternative routine test. Further studies are warranted to achieve the best strategy for management of childhood respiratory tract infections in Burkina Faso. More extensive research on the etiological agents of ARIs can only be beneficial, facilitating early adoption of appropriate treatment strategies.
